# Associations between pan-immune-inflammation value and abdominal aortic calcification: a cross-sectional study

**DOI:** 10.3389/fimmu.2024.1370516

**Published:** 2024-03-28

**Authors:** Chen Jin, Xunjia Li, Yuxiao Luo, Cheng Zhang, Deyu Zuo

**Affiliations:** ^1^ Department of Cardiothoracic Surgery, The First Affiliated Hospital of Chongqing Medical University, Chongqing, China; ^2^ Department of Nephrology, Chongqing Hospital of Traditional Chinese Medicine, Chongqing, China; ^3^ Chongqing Precision Medical Industry Technology Research Institute, Chongqing, China; ^4^ University Medical Center Göttingen, University of Göttingen, Göttingen, Germany; ^5^ Department of Rehabilitation Medicine, Chongqing Hospital of Traditional Chinese Medicine, Chongqing, China

**Keywords:** abdominal aortic calcification, pan-immune inflammation value, cardiovascular disease, NHANES, inflammation

## Abstract

**Background:**

Abdominal aortic calcification (AAC) pathogenesis is intricately linked with inflammation. The pan-immune-inflammation value (PIV) emerges as a potential biomarker, offering reflection into systemic inflammatory states and assisting in the prognosis of diverse diseases. This research aimed to explore the association between PIV and AAC.

**Methods:**

Employing data from the National Health and Nutrition Examination Survey (NHANES), this cross-sectional analysis harnessed weighted multivariable regression models to ascertain the relationship between PIV and AAC. Trend tests probed the evolving relationship among PIV quartiles and AAC. The study also incorporated subgroup analysis and interaction tests to determine associations within specific subpopulations. Additionally, the least absolute shrinkage and selection operator (LASSO) regression and multivariable logistic regression were used for characteristics selection to construct prediction model. Nomograms were used for visualization. The receiver operator characteristic (ROC) curve, calibration plot and decision curve analysis were applied for evaluate the predictive performance.

**Results:**

From the cohort of 3,047 participants, a distinct positive correlation was observed between PIV and AAC. Subsequent to full adjustments, a 100-unit increment in PIV linked to an elevation of 0.055 points in the AAC score (β=0.055, 95% CI: 0.014-0.095). Categorizing PIV into quartiles revealed an ascending trend: as PIV quartiles increased, AAC scores surged (β values in Quartile 2, Quartile 3, and Quartile 4: 0.122, 0.437, and 0.658 respectively; P for trend <0.001). Concurrently, a marked rise in SAAC prevalence was noted (OR values for Quartile 2, Quartile 3, and Quartile 4: 1.635, 1.842, and 2.572 respectively; P for trend <0.01). Individuals aged 60 or above and those with a history of diabetes exhibited a heightened association. After characteristic selection, models for predicting AAC and SAAC were constructed respectively. The AUC of AAC model was 0.74 (95%CI=0.71-0.77) and the AUC of SAAC model was 0.84 (95%CI=0.80-0.87). According to the results of calibration plots and DCA, two models showed high accuracy and clinical benefit.

**Conclusion:**

The research findings illuminate the potential correlation between elevated PIV and AAC presence. Our models indicate the potential utility of PIV combined with other simple predictors in the assessment and management of individuals with AAC.

## 1 Introduction

Abdominal arterial calcification (AAC) encompasses a complex process involving multiple cellular and molecular mechanisms. Arterial calcification occurs when arterial walls accrue deposits of calcium alongside other minerals, culminating in calcified plaque formations (1, 2). This intricate cascade is inherently tied to escalating cardiovascular risks, encapsulating diminished vascular elasticity, increased vascular stiffness, and atherosclerosis’ onset (3).

A hallmark of arterial calcification is its pronounced inflammatory signature. Various mediators, including cytokines and chemokines, facilitate the mobilization and activation of immune cells within arterial structures (4, 5). These cells secrete specific elements pivotal for the transformation of vascular smooth muscle cells (VSMCs) to osteoblast-like phenotypes, setting the stage for calcification (6–10).

Unfortunately, therapeutic strategies for preventing and relieving AAC remain elusive. Severe AAC can potentiate the risks of major adverse cardiovascular events and threaten patient prognosis. The necessity in clinical practice for early detection and routine screening with simple, cost-effective, and easily accessible tools, is pressing.

Recent investigations highlight the pan-immune-inflammation value (PIV) as an indicator of systemic inflammation status (11–13). Contrasting with to other immune indicators, such as he neutrophil-to-lymphocyte ratio (NLR), monocyte-to-lymphocyte ratio (MLR) and platelet-to-lymphocyte ratio (PLR), PIV emerges as potentially offering a more comprehensive reflection of inflammation. This is attributed to the calculation of PIV integrates counts of four principal immune cell types in the peripheral blood: neutrophils, monocytes, platelets, and lymphocytes (13, 14).

Existing literature scarcely explores the association between inflammation biomarkers and cardiovascular diseases, including AAC. Notably, there is no studies demonstrating the association between PIV and AAC. In this context, the present study endeavors to delineate the potential relationship between PIV and AAC.

Utilizing data from the 2013-2014 National Health and Nutrition Examination Survey (NHANES), we conducted a cross-sectional analysis, striving to elucidate the relationship between the PIV and AAC and to assess the applicability of PIV as a predictive marker for populations at risk of developing AAC.

## 2 Methods

### 2.1 Survey description and study population

The National Health and Nutrition Examination Survey (NHANES), conducted by the Centers for Disease Control and Prevention (CDC), constitutes a collection of surveys that are designed to record and evaluate the health and nutritional status of the U.S. population.

**Figure 1:**
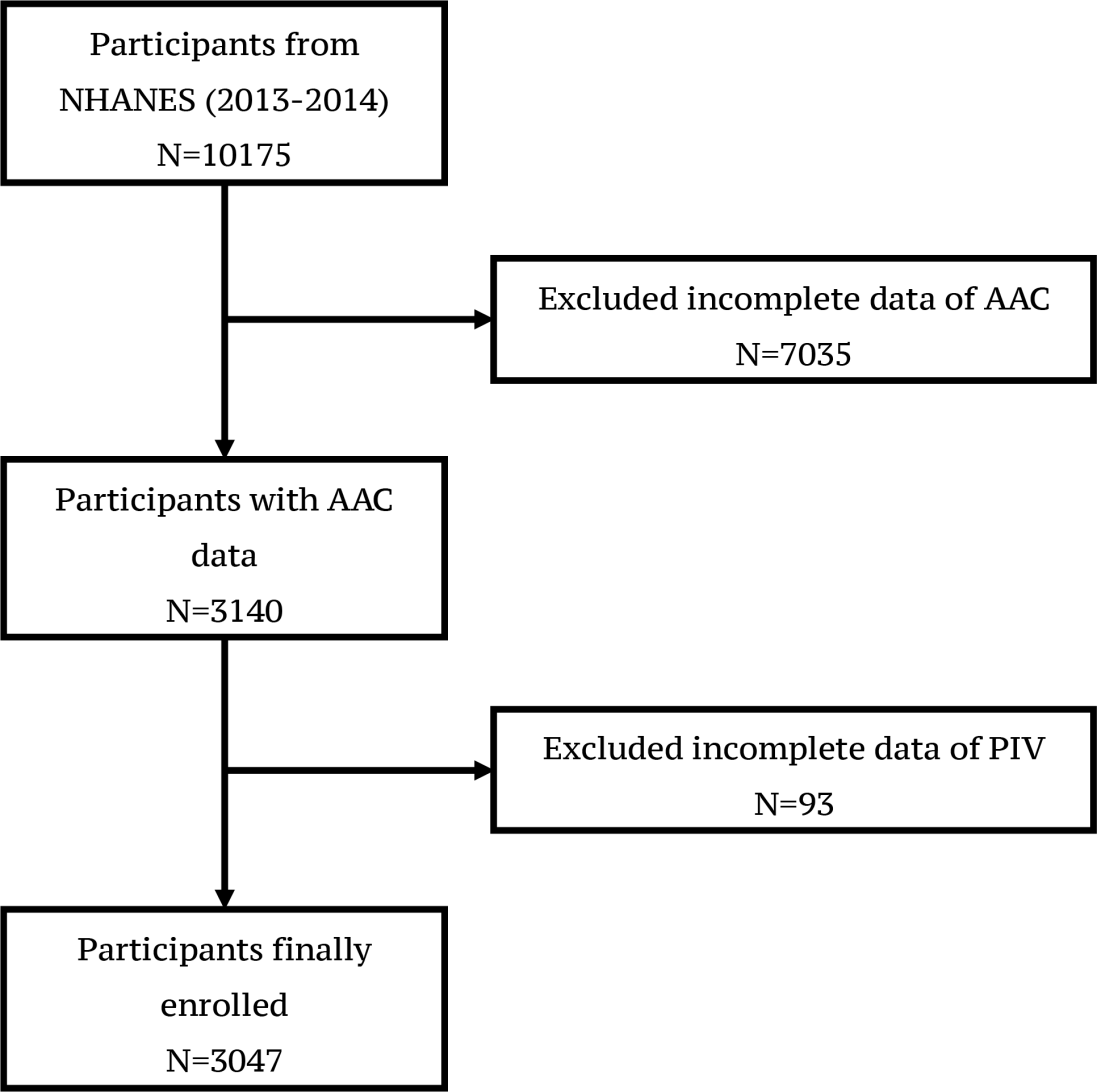
Flowchart of participants selection from NHANES (2013-2014) A total of 10175 participants were enrolled initially. 7128 participants were excluded due to missing or incomplete AAC and PIV data. 3047 participants were finally enrolled in the study.

In this study, an initial cohort of 10175 participants recruited from 2013 to 2014, was identified. During data wrangling and screening, 7128 participants were excluded due to missing or incomplete AAC and PIV data. Consequently, the study encompassed 3,047 participants aged ≥40 years (**Figure 1**).

### 2.2 Evaluation abdominal aortic calcification

Applying a dual-energy X-ray absorptiometry scan (DXA) and the Kauppila scoring system, the severity of abdominal aortic calcification was gauged in this study. AAC scores ranged from 0 to 24, with scores >6 regarding as severe AAC (SAAC). A higher AAC score indicates escalated calcification severity (15–17). The primary outcome variables were AAC score and severe AAC in this study. Scanning results for each participant were conducted using the Hologic Discovery model A densitometers (Hologic, Inc., Marlborough, Massachusetts). Detailed description about AAC measurement can be found at https://wwwn.cdc.gov/Nchs/Nhanes/2013-2014/DXXAAC_H.htm.

### 2.3 Assessment of pan-immune inflammation value

The pan-immune-inflammation value is calculated by neutrophil number × platelet number × monocyte number/lymphocyte number, with cell counts represented as ×1000 cells/uL. PIV/100 indicated PIV divided by 100. The blood test was performed by Beckman Coulter DxH 800 instrument in the NHANES mobile examination center.

### 2.4 Covariables

Based on previous investigations, we integrated confounders that may bias the association between PIV and AAC. The demographic variables included gender, age, race, and level of education. Laboratory and health-related factors were body mass index (BMI, kg/m²), waist circumference (cm), triglyceride (mmol/L), total cholesterol (mmol/L), serum Vitamin D (nmol/L), total calcium (mmol/L), history of alcohol consumption, hypertension, diabetes, asthma, celiac disease, arthritis, heart failure, heart attack, coronary heart disease, stroke, chronic obstructive pulmonary disease (COPD) and cancer. More details about these covariates can be found at: https://wwwn.cdc.gov/nchs/nhanes/continuousnhanes/default.aspx?BeginYear=2013.

### 2.5 Statistical analysis

Continuous variables were presented as mean ± standard deviation (SD), while categorical variables were represented as proportions. Weighted Student’s t-tests were used to assess differences among PIV quartiles for continuous variables, whereas weighted chi-square tests were applied for categorical variables. Weighted multivariable regression models were employed to assess the correlation between PIV and AAC. In the initial model no covariate was adjusted (crude model); in the partially adjusted model, gender, age, race and education level were adjusted. In the fully adjusted model, sex, age, race, education level, body mass index (BMI), waist circumference, average sagittal abdominal diameter, triglyceride, total cholesterol, serum Vitamin D, total calcium, history of alcohol consumption, hypertension, diabetes, asthma, celiac disease, arthritis, heart failure, coronary heart disease, heart attack, stroke, emphysema, COPD and cancer, were all adjusted. Trend tests probed the association among PIV quartiles. Subgroup analysis was conducted to evaluate the association between PIV and AAC by stratifying sex, age, BMI, alcohol consumption, diabetes, smoke, hypertension, coronary heart disease. Interaction tests were also applied to assess the associations within subgroups. Covariates in full adjusted model were included in subgroup analysis as well. The nonlinear association between PIV and AAC was evaluated by using smoothed curve fitting.

Variables for model construction were selected by the LASSO regression and the multivariable regression analysis. The nomogram was used for visualizing the model. The Model performance was assessed by using the ROC curve, the calibration curve and the decision curve analysis. R (version 4.2) was applied for all statistical analysis, with a statistical significance threshold set at a two-sided P<0.05.

## 3 Results

### 3.1 Baseline characteristics

This study incorporated a cohort of 3,047 participants, with a mean age of 58.63 ± 12.00 years (mean ± SD). Of these, 1469 (48.21%) were male and 1578 (51.79%) were female. The racial composition included Mexican Americans (13.19%), other Hispanics (9.48%), non-Hispanics (63.51%), and individuals of other races (13.82%). The average AAC score (mean ± SD) for all participants was 1.63 ± 3.51, and 273 (8.96%) participants were identified with severe AAC. Furthermore, the mean PIV for all participants was 315.22 ± 301.40 (mean ± SD). The PIV was segmented into quartiles, with Q1 (n=762) ≤156.22, Q2 (n=761) >156.22 and ≤242.15, Q3 (n=762) >242.15 and ≤ 372.53, and Q4 (n=762) > 372.53. A noteworthy trend revealed the ACC score rose with increasing PIV quartiles (Q1: 1.26 ± 2.89, Q2: 1.33 ± 2.99, Q3: 1.64 ± 3.60, and Q4: 2.30 ± 4.27 respectively, *P*<0.001). A similar trend was evident for SAAC prevalence among these quartiles (*P*<0.001) (**Table 1**).

**Table 1:** Baseline characteristics of participants by quartiles of pan-immune inflammation value.

Characteristics	Pan-immune inflammation value				*P* value
	Q1(N=762)	Q2(N=761)	Q3(N=762)	Q4(N=762)	
Age (year)	58.05 ± 11.36	58.02 ± 11.57	58.21 ± 12.06	60.24 ± 12.84	0.002
Sex (%)					0.085
Male	357 (46.85%)	359 (47.17%)	355 (46.59%)	398 (52.23%)	
Female	405 (53.15%)	402 (52.83%)	407 (53.41%)	364 (47.77%)	
Race (%)					<0.001
Mexican American	89 (11.68%)	105 (13.80%)	101 (13.25%)	107 (14.04%)	
Other Hispanic	73 (9.58%)	78 (10.25%)	88 (11.55%)	50 (6.56%)	
Non-Hispanic	454 (59.58%)	462 (60.71%)	480 (62.99%)	539 (70.73%)	
Other Race	146 (19.16%)	116 (15.24%)	93 (12.20%)	66 (8.66%)	
Alcohol (%)					<0.001
Yes	441 (62.82%)	527 (72.79%)	521 (72.97%)	553 (76.28%)	
No	261 (37.18%)	197 (27.21%)	193 (27.03%)	172 (23.72%)	
Smoke (%)					<0.001
Yes	314 (41.26%)	314 (41.26%)	349 (45.80%)	430 (56.43%)	
No	447 (58.74%)	447 (58.74%)	413 (54.20%)	332 (43.57%)	
Hypertension (%)					<0.001
Yes	329 (43.18%)	345 (45.45%)	356 (46.78%)	413 (54.27%)	
No	433 (56.82%)	414 (54.55%)	405 (53.22%)	348 (45.73%)	
Diabetes (%)					0.002
Yes	104 (13.65%)	119 (15.64%)	124 (16.27%)	159 (20.87%)	
No	658 (86.35%)	642 (84.36%)	638 (83.73%)	603 (79.13%)	
Asthma (%)					0.001
Yes	82 (10.78%)	86 (11.30%)	116 (15.24%)	126 (16.54%)	
No	679 (89.22%)	675 (88.70%)	645 (84.76%)	636 (83.46%)	
Arthritis (%)					0.008
Yes	243 (31.97%)	235 (30.96%)	275 (36.14%)	290 (38.21%)	
No	517 (68.03%)	524 (69.04%)	486 (63.86%)	469 (61.79%)	
Celiac disease (%)					0.602
Yes	7 (0.92%)	5 (0.66%)	3 (0.39%)	4 (0.53%)	
No	754 (99.08%)	756 (99.34%)	759 (99.61%)	757 (99.47%)	
Heart failure (%)					0.007
Yes	19 (2.49%)	22 (2.89%)	22 (2.91%)	41 (5.39%)	
No	743 (97.51%)	738 (97.11%)	735 (97.09%)	720 (94.61%)	
Coronary heart disease (%)					<0.001
Yes	26 (3.42%)	32 (4.22%)	40 (5.26%)	61 (8.04%)	
No	735 (96.58%)	727 (95.78%)	720 (94.74%)	698 (91.96%)	
Stroke (%)					0.003
Yes	27 (3.55%)	26 (3.43%)	27 (3.54%)	51 (6.69%)	
No	733 (96.45%)	733 (96.57%)	735 (96.46%)	711 (93.31%)	
Heart attack (%)					<0.001
Yes	28 (3.675%)	27 (3.557%)	34 (4.468%)	62 (8.136%)	
No	734 (96.325%)	732 (96.443%)	727 (95.532%)	700 (91.864%)	
COPD (%)					<0.001
Yes	23 (3.02%)	17 (2.23%)	31 (4.08%)	74 (9.71%)	
No	738 (96.98%)	744 (97.77%)	729 (95.92%)	688 (90.29%)	
Cancer (%)					0.037
Yes	82 (10.76%)	88 (11.56%)	93 (12.20%)	117 (15.35%)	
No	680 (89.24%)	673 (88.44%)	669 (87.80%)	645 (84.65%)	
BMI	27.73 ± 5.31	28.03 ± 5.26	28.73 ± 5.50	29.33 ± 6.07	<0.001
Waist circumference(cm)	96.62 ± 13.45	97.94 ± 13.05	100.04 ± 13.05	102.69 ± 14.42	<0.001
Triglyceride(mmol/L)	1.32 ± 1.12	1.36 ± 0.81	1.39 ± 0.90	1.46 ± 0.91	<0.001
Cholesterol mmol/L)	5.09 ± 1.06	5.116 ± 1.137	5.123 ± 1.118	4.939 ± 1.11	0.003
Vitamin D (nmol/L)	68.77 ± 29.42	71.52 ± 30.70	69.31 ± 28.24	71.96 ± 28.85	0.107
Serum calcium(mmol/L)	2.36 ± 0.09	2.36 ± 0.09	2.36 ± 0.09	2.37 ± 0.10	0.697
Neutrophil number	49.73 ± 8.51	56.21 ± 6.51	59.83 ± 6.72	66.25 ± 7.08	<0.001
Lymphocyte number	2.18 ± 0.80	2.13 ± 0.69	2.11 ± 0.76	1.95 ± 0.70	<0.001
Monocyte number	0.44 ± 0.13	0.53 ± 0.13	0.61 ± 0.15	0.75 ± 0.23	<0.001
Platelet number	198.12 ± 49.42	221.31 ± 49.89	238.71 ± 50.72	265.67 ± 65.14	<0.001
PIV	109.60 ± 31.20	197.84 ± 24.25	299.86 ± 37.36	653.06 ± 436.09	<0.001
AAC score	1.26 ± 2.89	1.33 ± 2.99	1.64 ± 3.60	2.30 ± 4.27	<0.001
SAAC (%)					<0.001
Yes	60 (7.87%)	45 (5.91%)	78 (10.24%)	90 (10.81%)	
No	702 (92.13%)	716 (94.09%)	684 (89.76%)	672 (88.19%)	

Continuous variables were described as mean ± SD. Categorical variables were shown as numbers and percentages (%). Q quartile, SD standard deviation, BMI body mass index, COPD chronic obstructive pulmonary disease, AAC abdominal aortic calcification, SAAC severe abdominal aortic calcification. PIV pan-immune-inflammation value. The units of neutrophil, lymphocyte, monocyte and platelet number were 1000 cells/uL.

### 3.2 Association between higher PIV and increased AAC scores and SAAC incidence

**Figure 2:**
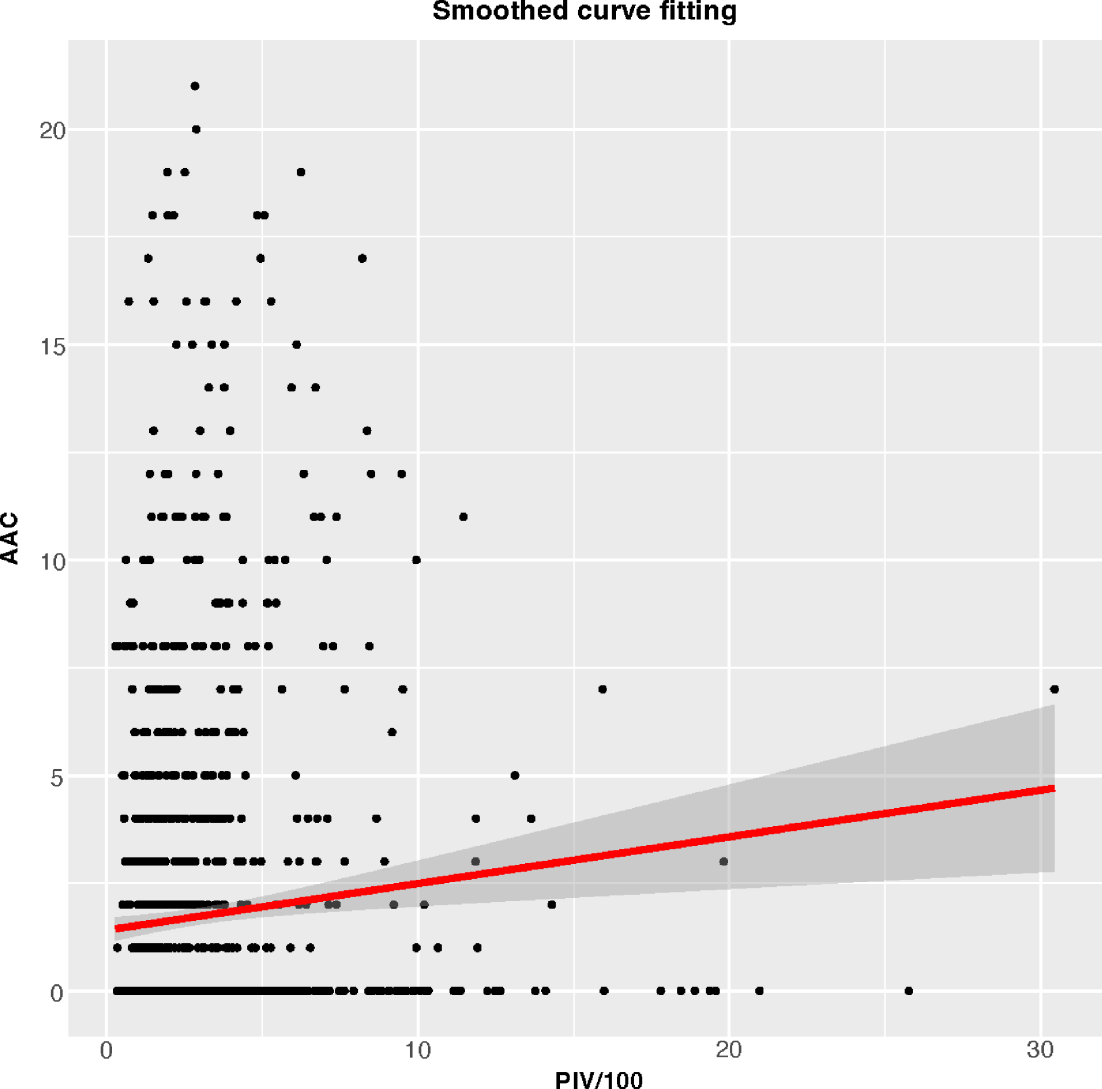
Smoothed curve fitting for PIV/100 and AAC.

**Table 2** depicted the association between PIV and AAC. In the crude model (β=0.110, 95%CI= 0.069-0.151), partially adjusted model (β=0.077, 95%CI=0.039-0.115), and fully adjusted model (β=0.055, 95%CI=0.014-0.095), it was discovered that a higher PIV associated with higher AAC scores. After full adjustment, the AAC score increased by 0.055 points for every 100-unit increment in PIV. Upon categorizing PIV into quartiles, the β values for Quartiles 2, 3, and 4, relative to the lowest PIV quartile, were 0.122, 0.437, and 0.658 respectively. (Quartile 2: β=0.122, 95%CI= -0.208-0.453; Quartile 3: β=0.437, 95%CI=0.103-0.771; Quartile 4: β=0.658, 95%CI=0.319-0.998; *P* for trend <0.001). Additionally, a potential correlation between PIV and SAAC incidence emerged. The results revealed an augmented SAAC incidence with elevated PIV across different models: crude model (OR=1.034, 95%CI=1.004-1.067), partially adjusted model (OR=1.039, 95%CI=1.007-1.073), and fully adjusted model (OR=1.050, 95%CI=0.985-1.120). Intriguingly, the risk for SAAC surged by 157.2% for individuals in the highest PIV quartile compared to their counterparts in the lowest quartiles in the fully adjusted model (*P* for trend <0.001). The nonlinear association between PIV and AAC was further corroborated through smoothed curve fitting (**Figure 2**).

**Table 2:** Associations between pan-immune inflammation value and abdominal aortic calcification.

PIV	AAC Score	Severe AAC
	β (95% CI)	OR (95% CI)
Crude model (Model 1) ^a^		
Continuous PIV	0.110 (0.069, 0.151) ***	1.035 (1.004, 1.067) 0.02566
Categories PIV		
Quartile 1	0 (refence)	1 (refence)
Quartile 2	0.067 (-0.283, 0.417)	0.889 (0.620, 1.276)
Quartile 3	0.386 (0.036, 0.736) *	1.384 (0.993, 1.928)
Quartile 4	1.041 (0.691, 1.390) ***	1.704 (1.236, 2.349) **
*P* for tend	0.264 (0.184, 0.344) ***	1.160 (1.080, 1.246) ***
Partially adjusted model (Model 2) ^b^		
Continuous PIV	0.077 (0.039, 0.115) ***	1.039 (1.007, 1.073) *
Categories PIV		
Quartile 1	0 (refence)	1 (refence)
Quartile 2	0.082 (-0.240, 0.403)	0.853 (0.591, 1.230)
Quartile 3	0.383 (0.060, 0.705) *	1.310 (0.935, 1.835)
Quartile 4	0.789 (0.464, 1.113) ***	1.657 (1.193, 2.301) **
P for tend	0.198 (0.124, 0.273) ***	1.157 (1.076, 1.246) ***
Fully adjusted model (Model 3) ^c^		
Continuous PIV	0.0547 (0.0144, 0.0950) **	1.050 (0.985, 1.120)
Categories PIV		
Quartile 1	0 (refence)	1 (refence)
Quartile 2	0.122 (-0.208, 0.453)	1.635 (0.873, 3.064)
Quartile 3	0.437 (0.103, 0.771) *	1.842 (1.003, 3.385) *
Quartile 4	0.658 (0.319, 0.998) ***	2.572 (1.411, 4.689) **
P for tend	0.162 (0.085, 0.240) ***	1.216 (1.070, 1.382) **

a Crude model (Model 1): no covariate was adjusted.

b Partially adjusted model (Model 2): sex, age, race and education level were adjusted.

c Fully adjusted model was adjusted for sex, age, race, education level, BMI, waist circumference, triglyceride, total cholesterol, serum Vitamin D, total calcium, history of alcohol consumption, hypertension, diabetes, asthma, celiac disease, arthritis, heart failure, heart attack, coronary heart disease, stroke, COPD and cancer. *P<0.05, ** P<0.01, ***P<0.001. CI confidence interval, PIV pan-immune inflammation value, AAC abdominal aortic calcification, BMI body mass index, COPD chronic obstructive pulmonary disease.

### 3.3 Subgroup analysis

To further examine the consistency of the association between PIV and AAC across the entire population and discern any potential disparities in specific subpopulations, we carried out subgroup analysis and interaction tests. Stratifications were conducted including gender, age, BMI, and medical histories (alcohol consumption, smoke, diabetes, hypertension and coronary heart disease) (**Table 3**). Our findings revealed significant interaction effects between age and diabetes history with respect to their association between PIV and AAC score. Explicitly, in the elderly (≥60 years) and those with a diabetes history, AAC scores increased by 0.114 and 0.188 points per 100-unit increase in PIV respectively. However, no such interactions were identified for severe AAC. The other stratifying factors did not indicate significant interactions with the association.

**Table 3:** Subgroup analysis of the association between PIV and AAC.

Subgroup	ACC score		Severe ACC	
	β (95% CI)	*P* for interaction	OR (95% CI)	*P* for interaction
Sex		0.254		0.216
Male	0.003 (-0.097, 0.103)		0.935 (0.729, 1.200)	
Female	0.079 (-0.011, 0.168)		1.115 (0.971, 1.280)	
Age		0.031		0.574
<60	-0.029 (-0.125, 0.067)		1.027 (0.854, 1.234)	
≥60	0.114 (0.021, 0.206) *		1.099 (0.941, 1.284)	
BMI		0.474		0.999
<25	0.089 (-0.053, 0.232)		0.931 (0.023, 44.148)	
≥25	0.032 (-0.044, 0.107)		1.012 (0.910, 1.126)	
Alcohol consumption		0.657		0.655
Yes	0.052 (-0.023, 0.128)		1.041 (0.883, 1.227)	
No	0.016 (-0.131, 0.163)		1.100 (0.923, 1.310)	
Smoke		0.604		0.681
Yes	0.033 (-0.051, 0.116)		1.091 (0.935, 1.272)	
No	0.070 (-0.046, 0.185)		1.037 (0.862, 1.246)	
Diabetes		0.032		0.616
Yes	0.188 (0.040, 0.335) *		1.008 (0.777, 1.308)	
No	0.010 (-0.065, 0.085)		1.085 (0.949, 1.241)	
Hypertension		0.993		0.956
Yes	0.045 (-0.039, 0.130)		1.065 (0.910, 1.246)	
No	0.045 (-0.064, 0.154)		1.072 (0.894, 1.285)	
Coronary heart disease		0.301		0.873
Yes	-0.059 (-0.270, 0.152)		1.015 (0.514, 2.005)	
No	0.056 (-0.015, 0.128)		1.070 (0.947, 1.208)	

In subgroup analysis, covariates of sex, age, race, education level, BMI, waist circumference, triglyceride, total cholesterol, serum Vitamin D, total calcium, history of alcohol consumption, hypertension, diabetes, asthma, celiac disease, arthritis, heart failure, heart attack, coronary heart disease, stroke, COPD and cancer were adjusted. *P<0.05. CI, confidence interval; PIV, pan-immune inflammation value; AAC, abdominal aortic calcification; BMI, body mass index; COPD, chronic obstructive pulmonary disease.

### 3.4 LASSO regression analysis for characteristics screening

**Figure 3:**
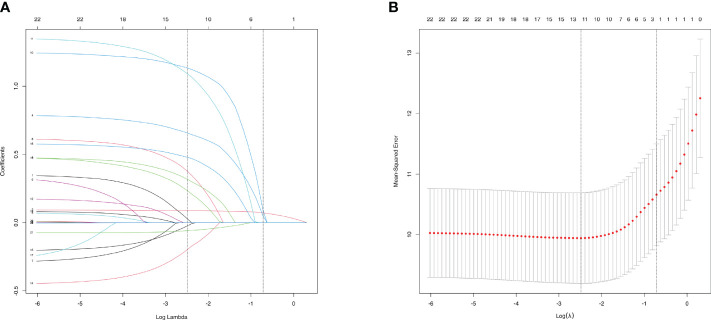
Characteristics Screening for AAC prediction by Lasso regression. **(A)**. Regression coefficient profile diagram. Each curve represents the change trajectory of each characteristic coefficient. **(B)**. Cross-validation curve of Lasso regression. Each red dots represents Mean-squared error (MSE) for each value of λ. The ordinate is the value of the coefficient, the abscissa (upper) is the number of non-zero coefficients in the model, the abscissa (lower) is logarithmic value of the regularization parameter λ. Dashed line on the left shows the minimum value of lambda (lambda.min, log(λ) = -2.48) and the dashed line (right) shows the value of one standard error of lambda (lambda.1se, log(λ) = -0.72). For AAC prediction, we used the lambda.min for variable screening. Twelve characteristics were selected: Gender, Age, Smoke, Heart failure, Coronary heart disease, Heart attack, Stroke, Cancer, Alcohol, Diabetes, Hypertension and BMI.

**Figure 4:**
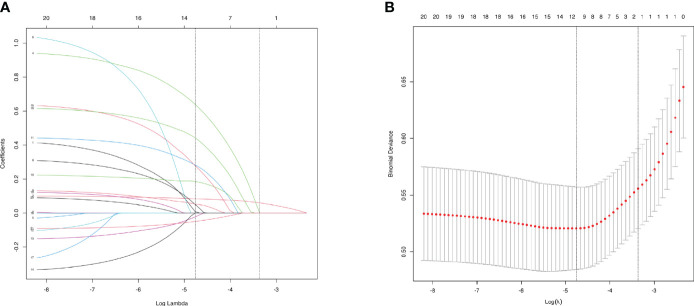
Characteristics Screening for SAAC prediction by Lasso regression. **(A)**. Regression coefficient profile diagram. Each curve represents the change trajectory of each characteristic coefficient. **(B)**. Cross-validation curve of Lasso regression. Each red dots represents Mean-squared error (MSE) for each value of λ. The ordinate is the value of the coefficient, the abscissa (upper) is the number of non-zero coefficients in the model, the abscissa (lower) is logarithmic value of the regularization parameter λ. Dashed line on the left shows the minimum value of lambda (lambda.min, log(λ) = -4.76) and the dashed line (right) shows the value of one standard error of lambda (lambda.1se, log(λ) = -3.36). For SAAC prediction, we used the lambda.min for variable screening. Eleven characteristics were selected: Gender, Age, Smoke, Heart failure, Coronary heart disease, Heart attack, Stroke, Cancer, Diabetes, Hypertension and BMI.

To select characteristics that can be applied to predict incidence of AAC and SAAC, LASSO regression analysis was performed on variables in the baseline characteristics, besides PIV. For AAC prediction, twelve characteristics: Gender, Age, Smoke, Heart failure, Coronary heart disease, Heart attack, Stroke, Cancer, Alcohol, Diabetes, Hypertension and BMI were selected with minimum value of λ (**Figure 3**). For SAAC prediction, other 11 characteristics: Gender, Age, Smoke, Celiac disease, Heart failure, Coronary heart disease, Heart attack, Stroke, Alcohol, Diabetes, Hypertension, Triglyceride and BMI were selected with minimum value of λ (**Figure 4**).

### 3.5 Multivariable logistic regression for characteristics selection

To construct models for predicting the incidences of AAC and SAAC, multivariable logistic regression analysis was applied to further select the results based on Lasso regression.

For AAC prediction model, the following characteristics significantly associated with AAC were included in further ROC curve to diagnose AAC: Age (OR=1.098, 95%CI=1.080-1.115, *P* value<0.001), Smoke (OR= 2.147, 95%CI=1.495-3.084, *P* value<0.001), Heart attack (OR=3.567, 95%CI=1.457-8.734, *P* value=0.005), Stroke (OR=3.957, 95%CI=1.731-9.048, *P* value=0.001), Alcohol (OR=1.571, 95%CI=1.048-2.355, *P* value=0.029), Hypertension (OR=1.612, 95%CI=1.109-2.343, *P* value=0.012), and BMI (OR=1.580, 95%CI=1.070-2.332, *P* value=0.021) (**Table 4**). For SAAC prediction model, the following characteristics were selected for further ROC curve to diagnose SAAC: Age (OR=1.101, 95%CI=1.077-1.125, *P* value<0.001), Smoke (OR=2.590, 95%CI=1.689-3.971, *P* value<0.001), Hypertension (OR=1.790, 95%CI=1.141-2.806, *P* value=0.011) and BMI (OR=1.599, 95%CI=1.029-2.486, *P* value=0.037) (**Table 5**).

**Table 4:** Multivariate logistic regression for characteristics selection in the AAC model.

Characteristics	Estimate	SE	OR	95% CI	*P* value
**Intercept**	-5.163	0.514	0.006	0.002, 0.016	<0.001
**Gender**	0.224	0.183	1.251	0.873, 1.792	0.222
**Age**	0.093	0.008	1.098	1.080, 1.115	<0.001
**Smoke**	0.764	0.185	2.147	1.495, 3.084	<0.001
**Heart failure**	0.515	0.496	1.674	0.633, 4.425	0.299
**Coronary heart disease**	0.493	0.459	1.637	0.666, 4.023	0.283
**Heart attack**	1.272	0.457	3.567	1.457, 8.734	0.005
**Stroke**	1.376	0.422	3.957	1.731, 9.048	0.001
**Cancer**	0.188	0.263	1.207	0.721, 2.021	0.474
**Alcohol**	0.452	0.206	1.571	1.048, 2.355	0.029
**Diabetes**	0.357	0.249	1.430	0.878, 2.327	0.151
**Hypertension**	0.477	0.191	1.612	1.109, 2.343	0.012
**BMI**	0.457	0.199	1.580	1.070, 2.332	0.021

Twelve characteristics were screened from previous LASSO regression. Then, characteristics with P<0.05 in the multivariate logistic regression analysis were included in the further model, which were Age, Smoke, Heart attack, Stroke, Alcohol, Hypertension and BMI.

**Table 5:** Multivariate logistic regression for characteristics selection in the SAAC model.

Characteristics	Estimate	SE	OR	95% CI	*P* value
**Intercept**	-9.835	0.814	0.0001	0.0001, 0.0002	<0.001
**Gender**	0.284	0.210	1.329	0.881, 2.003	0.175
**Age**	0.096	0.011	1.101	1.077, 1.125	<0.001
**Smoke**	0.952	0.218	2.590	1.689, 3.971	<0.001
**Heart failure**	0.155	0.411	1.168	0.521, 2.615	0.706
**Coronary heart disease**	0.098	0.381	1.103	0.522, 2.327	0.798
**Heart attack**	0.282	0.377	1.326	0.633, 2.776	0.455
**Stroke**	0.448	0.344	1.566	0.798, 3.073	0.193
**Cancer**	0.187	0.242	1.205	0.750, 1.937	0.441
**Diabetes**	0.461	0.246	1.586	0.979, 2.570	0.061
**Hypertension**	0.582	0.230	1.790	1.141, 2.806	0.011
**BMI**	0.470	0.225	1.599	1.029, 2.486	0.037

Eleven characteristics were screened from previous LASSO regression. Then, characteristics with P<0.05 in the multivariate logistic regression analysis were selected in the further model, which were Age, Smoke, Diabetes, Hypertension and BMI.

### 3.6 Nomogram development for risk prediction

**Figure 5:**
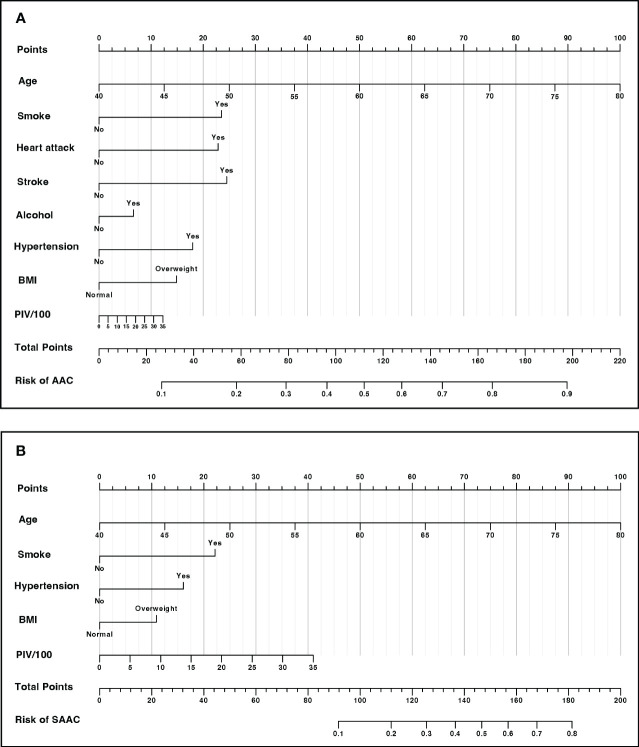
Nomogram for risk prediction **(A)** Nomogram for AAC prediction. **(B)** Nomogram for SAAC prediction.

After two steps of characteristics selection (LASSO regression and multivariable logistic regression), the nomogram was constructed for providing a visual tool used for predicting the risk of incidences of AAC and SAAC. In the final model, characteristics in prediction model for AAC were PIV, Age, Smoke, Heart attack, Stroke, Alcohol, Hypertension and BMI (**Figure 5A**). In the SAAC model, the following characteristics were involved: PIV, Age, Smoke, Hypertension and BMI (**Figure 5B**).

### 3.7 ROC curves for the incidences of AAC and SAAC

**Figure 6:**
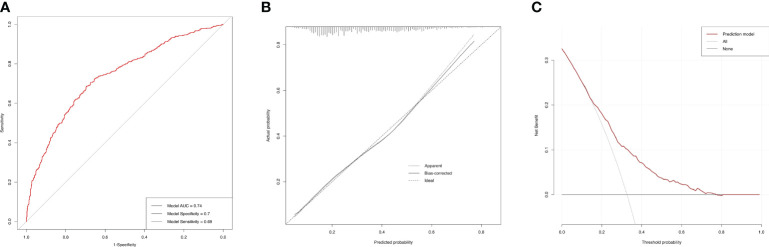
Performance evaluation of the AAC prediction model. **(A)** ROC curve. The AUC of the model was 0.74 (95% CI=0.71-0.77). The specificity and sensitivity of the model were 0.70 and 0.69 respectively. **(B)** Calibration Plot. The solid line for bias-corrected prediction, the grey dotted line for apparent prediction and the black dotted line for Ideal prediction. **(C)** Decision curve analysis (DCA). The red line for net benefit of the prediction model. The black line for no prediction model used.

**Figure 7:**
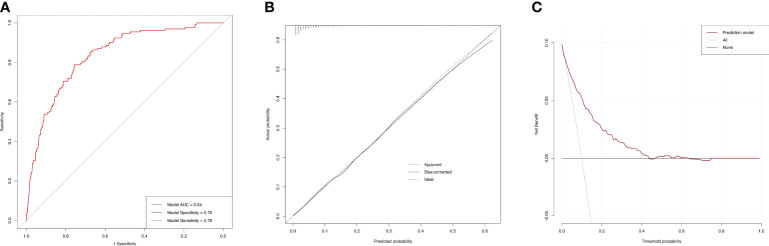
Performance evaluation of the SAAC prediction model. **(A)** ROC curve. The AUC of the model was 0.84 (95% CI=0.80-0.87). The specificity and sensitivity of the model were 0.75 and 0.79 respectively. **(B)** Calibration Plot. The solid line for bias-corrected prediction, the grey dotted line for apparent prediction and the black dotted line for Ideal prediction. **(C)** Decision curve analysis (DCA). The red line for net benefit of the prediction model. The black line for no prediction model used.

Based on the final model, the AUC of the AAC model was 0.74 (95%CI=0.71-0.77), and the specificity and sensitivity were 0.70 and 0.69, respectively (**Figure 6A**), which indicated good performance to identify AAC cases. The calibration plot showed the agreement between predicted probabilities and observed outcomes, which suggested high predictive accuracy (**Figure 6B**). The decision curve analysis (DCA) represented that the prediction model was beneficial within thresholds of probability (**Figure 6C**). In the model for SAAC, the AUC of the model was 0.84 (95%CI=0.80-0.87), and the specificity and sensitivity were 0.75 and 0.79, respectively (**Figure 7A**), which suggested good performance to identify SAAC cases. The calibration plot and DCA also indicated high predictive accuracy and decision benefit of the model (**Figures 7B, C**).

## 4 Discussion

In this cross-sectional study, a total of 3047 participants were in enrolled based on the NHANES database. The main findings of this study are as follow: 1) the association between PIV and AAC score was found a positive correlation. Higher PIV quartile was associated with higher AAC score and severe AAC incidence. 2) older and diabetes participants may potentially result in a higher risk of AAC or SAAC. 3) combining PIV and other easily-accessible factors, we developed prediction models for AAC and SAAC, which showed high accuracy and clinical benefit. These results point towards the potential utility of the model as a simple and easily-accessible tool to evaluate the incidence and severity of AAC.

To our best knowledge, this is the first study with a large-scale epidemiological analysis to elucidates the association between PIV and abdominal aortic calcification. The calculation of the pan-immune-inflammation value incorporates neutrophils, platelets, monocytes and lymphocytes, which are predominant immune cell type in peripheral blood and potentially reflect the systemic inflammatory conditions. Recent years, the focus of PIV-related research has mainly centered on its implications for the prognosis and therapeutic outcomes in oncological patients (11, 12, 18–23). For example, Zhai et al. found PIV as an independent prognostic indicator for NSCLC patients who achieved pathological complete response after receiving neoadjuvant immunochemotherapy (19). Meanwhile, Provenzano et al. retrospectively analyzed the association between increased PIV and adverse outcomes, including worse overall survival (HR: 4.46, 95%CI: 2.22-8.99) and progression-free survival (HR: 2.03, 95% CI: 1.08-3.80), besides, resistance to platinum-based chemotherapy in patients with advanced triple-negative breast cancer (20). Furthermore, PIV has also been discovered as a novel biomarker in reflecting the association between inflammation and noncancer diseases, such as in hypertension, heart failure, myocardial infarction, kidney disease, frailty, and hepatic steatosis (13, 24–28). Similarly, elevated PIV level correlated with the presence and severity of these noncancerous conditions.

Potential underlying mechanisms suggest that immunes cells play a significant role in these inflammatory diseases. Observations from our study also reinforce the prevailing understanding that systemic inflammation is central to the initiation and progression of vascular calcification. This aligns with an increase of literature emphasizing the complex relationship between inflammation and vascular calcification (10, 29, 30). A primary mechanism by which inflammation affects vascular calcification pertains to its modulation of VSMCs (31). These transformed VSMCs are instrumental in calcium deposition within arterial walls, a fundamental process in the pathogenesis of vascular calcification (1, 3, 31).

Several inflammatory mediators, including interleukin-6 (IL-6), IL-8,IL-29 and tumor necrosis factor-alpha (TNF-α), and transforming growth factor‐β (TGF-β), can induce phenotypic alterations in VSMCs. Such changes foster their differentiation into osteoblast-like cells (7–9, 31, 32). In the context of inflammation, neutrophils and monocytes release inflammatory mediators that can trigger dysfunction and apoptosis in VSMCs and endothelial cells (1, 4, 33). Platelets and platelet-derived factors, in their interaction with immune cells and endothelial cells, amplify the inflammatory environment within arteries (34, 35). Subsequently, these cells release matrix vesicles rich in calcium and phosphate, serving as potential focal points for calcium crystallization and fostering calcified deposit formation (36, 37). Lymphocytes, especially regulatory T cells (Tregs), may offer a protective influence against vascular calcification. This protection is largely attributed to their secretion of anti-inflammatory cytokines, notably IL-10, which acts to attenuate the activity of effector cells. Furthermore, Tregs demonstrate an inherent ability to impede the calcification process in VSMCs through the secretion of anti-calcific factors such as osteoprotegerin (OPG) (3, 31).

Abdominal aortic calcification stands as a potent harbinger of adverse cardiovascular events, including myocardial infarction and stroke. Hence, discerning and targeting systemic inflammation in AAC development could pave the way for therapeutic interventions and reducing the associated cardiovascular morbidity and mortality. Our findings presenting the association between PIV and AAC is profound in clinical practice. Certainly, other medical conditions such as lifestyle and disease history can exert an impact on the occurrence and progression of AAC. This influence has also been suggested by our multivariate regression model. Consequently, we have incorporated these easily-accessible clinical indicators associated with AAC occurrence and progression into our predictive model, which is aimed at facilitating the effective evaluation and management of high-risk patients.

However, it is essential to acknowledge the inherent limitations of our study. The cross-sectional design precludes the establishment of a definitive causal relationship between PIV and AAC. Future longitudinal investigations and randomized controlled trials are imperative to elucidate the causative mechanisms and to assess the efficacy of anti-inflammatory interventions. Besides, the dynamic fluctuation of systemic inflammation during the progression of AAC requires exploration through longitudinal investigations. The predictive model also needs a lager external cohort validation. Owing to constraints of the NHANES database, it is challenging to include all relevant covariates that might impact cardiovascular and inflammatory conditions. Other covariates, which are not included in our current model but may also demonstrate predictive potential, warrant further investigation. Given the scope of the datasets we analyzed, our findings may currently be applicable to a limited population, therefore, further prospective and multicenter studies are demanded.

## 5 Conclusion

Our study has highlighted the pan-immune-inflammation value establishing a robust association with both the occurrence and severity of AAC. PIV and other easily-accessible factors could feasibly function as a simple model for the assessing and managing individuals with AAC.

## Data availability statement

The original contributions presented in the study are included in the article/supplementary material. Further inquiries can be directed to the corresponding authors.

## Ethics statement

The studies involving humans were approved by The US Centers for Disease Control and Prevention. The studies were conducted in accordance with the local legislation and institutional requirements. The participants provided their written informed consent to participate in this study.

## Author contributions

CJ: Conceptualization, Data curation, Formal analysis, Investigation, Methodology, Validation, Visualization, Writing – original draft, Writing – review & editing. XL: Conceptualization, Data curation, Formal analysis, Writing – original draft, Writing – review & editing. YL: Data curation, Formal analysis, Methodology, Validation, Writing – original draft, Writing – review & editing. CZ: Funding acquisition, Project administration, Supervision, Validation, Writing – review & editing. DZ: Conceptualization, Funding acquisition, Project administration, Supervision, Writing – review & editing.

## References

[1] Wu M Rementer C Giachelli CM. Vascular calcification: an update on mechanisms and challenges in treatment. Calcified Tissue Int. (2013) 93:365–73. doi: 10.1007/s00223-013-9712-z PMC3714357 23456027

[2] Abedin M Tintut Y Demer LL. Vascular calcification: mechanisms and clinical ramifications. Arterioscler Thromb Vasc Biol. (2004) 24:1161–70. doi: 10.1161/01.ATV.0000133194.94939.42 15155384

[3] Johnson RC Leopold JA Loscalzo J. Vascular calcification: pathobiological mechanisms and clinical implications. Circ Res. (2006) 99:1044–59. doi: 10.1161/01.RES.0000249379.55535.21 17095733

[4] Passos LSA Lupieri A Becker-Greene D Aikawa E. Innate and adaptive immunity in cardiovascular calcification. Atherosclerosis. (2020) 306:59–67. doi: 10.1016/j.atherosclerosis.2020.02.016 32222287 PMC7483874

[5] Meng X Yang J Dong M Zhang K Tu E Gao Q. Regulatory T cells in cardiovascular diseases. Nat Rev Cardiol. (2016) 13:167–79. doi: 10.1038/nrcardio.2015.169 PMC11849084 26525543

[6] Borland SJ Morris TG Borland SC Morgan MR Francis SE Merry CLR. Regulation of vascular smooth muscle cell calcification by syndecan-4/FGF-2/PKCα signalling and cross-talk with TGFβ. Cardiovasc Res. (2017) 113:1639–52. doi: 10.1093/cvr/cvx178 PMC5852548 29016732

[7] Guerrero F Herencia C Almadén Y Martínez-Moreno JM Montes de Oca A Rodriguez-Ortiz ME. TGF-β prevents phosphate-induced osteogenesis through inhibition of BMP and Wnt/β-catenin pathways. PloS One. (2014) 9:e89179. doi: 10.1371/journal.pone.0089179 24586576 PMC3937350

[8] Hao N Zhou Z Zhang F Li Y Hu R Zou J. Interleukin-29 accelerates vascular calcification *via* JAK2/STAT3/BMP2 signaling. J Am Heart Assoc. (2023) 12:e027222. doi: 10.1161/JAHA.122.027222 36537334 PMC9973608

[9] Tintut Y Patel J Parhami F Demer LL. Tumor necrosis factor-alpha promotes in *vitro* calcification of vascular cells *via* the cAMP pathway. Circulation. (2000) 102:2636–42. doi: 10.1161/01.CIR.102.21.2636 11085968

[10] Sánchez-Cabo F Fuster V Silla-Castro JC González G Lorenzo-Vivas E Alvarez R. Subclinical atherosclerosis and accelerated epigenetic age mediated by inflammation: a multi-omics study. Eur Heart J. (2023) 44:2698–709. doi: 10.1093/eurheartj/ehad361 PMC10393076 37339167

[11] Yang X-C Liu H Liu D-C Tong C Liang X-W Chen R-H. Prognostic value of pan-immune-inflammation value in colorectal cancer patients: A systematic review and meta-analysis. Front Oncol. (2022) 12:1036890. doi: 10.3389/fonc.2022.1036890 36620576 PMC9813847

[12] Fucà G Guarini V Antoniotti C Morano F Moretto R Corallo S. The Pan-Immune-Inflammation Value is a new prognostic biomarker in metastatic colorectal cancer: results from a pooled-analysis of the Valentino and TRIBE first-line trials. Br J Cancer. (2020) 123:403–9. doi: 10.1038/s41416-020-0894-7 PMC7403416 32424148

[13] Murat B Murat S Ozgeyik M Bilgin M. Comparison of pan-immune-inflammation value with other inflammation markers of long-term survival after ST-segment elevation myocardial infarction. Eur J Clin Invest. (2023) 53:e13872. doi: 10.1111/eci.13872 36097823

[14] Liu Y Liu J Liu L Cao S Jin T Chen L. Association of systemic inflammatory response index and pan-immune-inflammation-value with long-term adverse cardiovascular events in ST-segment elevation myocardial infarction patients after primary percutaneous coronary intervention. J Inflamm Res. (2023) 16:3437–54. doi: 10.2147/JIR.S421491 PMC10438435 37600225

[15] Qin Z Du D Li Y Chang K Yang Q Zhang Z. The association between weight-adjusted-waist index and abdominal aortic calcification in adults aged ≥ 40 years: results from NHANES 2013-2014. Sci Rep. (2022) 12:20354. doi: 10.1038/s41598-022-24756-8 36437292 PMC9701694

[16] Qin Z Liu Q Jiao P Geng J Liao R Su B. Higher blood cadmium concentration is associated with increased likelihood of abdominal aortic calcification. Front In Cardiovasc Med. (2022) 9:870169. doi: 10.3389/fcvm.2022.870169 35557529 PMC9086707

[17] Liu N Feng Y Zhan Y Ma F. Relationship between blood cadmium and abdominal aortic calcification: NHANES 2013-2014. J Trace Elements In Med Biol Organ Soc For Minerals Trace Elements (GMS). (2022) 72:126975. doi: 10.1016/j.jtemb.2022.126975 35344900

[18] Corti F Lonardi S Intini R Salati M Fenocchio E Belli C. The Pan-Immune-Inflammation Value in microsatellite instability-high metastatic colorectal cancer patients treated with immune checkpoint inhibitors. Eur J Cancer (Oxford Engl 1990). (2021) 150:155–67. doi: 10.1016/j.ejca.2021.03.043 33901794

[19] Zhai W-Y Duan F-F Lin Y-B Lin Y-B Zhao Z-R Wang J-Y. Pan-immune-inflammatory value in patients with non-small-cell lung cancer undergoing neoadjuvant immunochemotherapy. J Inflamm Res. (2023) 16:3329–39. doi: 10.2147/JIR.S418276 PMC10422963 37576157

[20] Provenzano L Lobefaro R Ligorio F Zattarin E Zambelli L Sposetti C. The pan-immune-inflammation value is associated with clinical outcomes in patients with advanced TNBC treated with first-line, platinum-based chemotherapy: an institutional retrospective analysis. Ther Adv Med Oncol. (2023) 15:17588359231165978. doi: 10.1177/17588359231165978 37063779 PMC10102956

[21] Feng J Wang L Yang X Chen Q Cheng X. Clinical utility of preoperative pan-immune-inflammation value (PIV) for prognostication in patients with esophageal squamous cell carcinoma. Int Immunopharmacol. (2023) 123:110805. doi: 10.1016/j.intimp.2023.110805 37591121

[22] Gasparri ML Albasini S Truffi M Favilla K Tagliaferri B Piccotti F. Low neutrophil-to-lymphocyte ratio and pan-immune-inflammation-value predict nodal pathologic complete response in 1274 breast cancer patients treated with neoadjuvant chemotherapy: a multicenter analysis. Ther Adv Med Oncol. (2023) 15:17588359231193732. doi: 10.1177/17588359231193732 37720495 PMC10504832

[23] Yeh C-C Kao H-K Huang Y Tsai T-Y Young C-K Hung S-Y. Discovering the clinical and prognostic role of pan-immune-inflammation values on oral cavity squamous cell carcinoma. Cancers. (2023) 15. doi: 10.3390/cancers15010322 PMC9818418 36612318

[24] Demiröz Taşolar S Çiftçi N. Role of pan immune inflammatory value in the evaluation of hepatosteatosis in children and adolescents with obesity. J Pediatr Endocrinol Metab JPEM. (2022) 35:1481–6. doi: 10.1515/jpem-2022-0494 36284505

[25] Zhang F Li L Wu X Wen Y Zhan X Peng F. Pan-immune-inflammation value is associated with poor prognosis in patients undergoing peritoneal dialysis. Renal Fail. (2023) 45:2158103. doi: 10.1080/0886022X.2022.2158103 PMC9848369 36632816

[26] Okyar Baş A Güner M Ceylan S Hafızoğlu M Şahiner Z Doğu BB. Pan-immune inflammation value; a novel biomarker reflecting inflammation associated with frailty. Aging Clin Exp Res. (2023) 35:1641–9. doi: 10.1007/s40520-023-02457-0 37289361

[27] Wu B Zhang C Lin S Zhang Y Ding S Song W. The relationship between the pan-immune-inflammation value and long-term prognoses in patients with hypertension: National Health and Nutrition Examination Study, 1999-2018. Front Cardiovasc Med. (2023) 10:1099427. doi: 10.3389/fcvm.2023.1099427 36937901 PMC10017977

[28] Inan D Erdogan A Pay L Genc D Demırtola AI Yıldız U. The prognostic impact of inflammation in patients with decompensated acute heart failure, as assessed using the pan-immune inflammation value (PIV). Scandinavian J Clin Lab Invest. (2023) 83(6):371–8. doi: 10.1080/00365513.2023.2233890 37432669

[29] Xie R Liu X Wu H Liu M Zhang Y. Associations between systemic immune-inflammation index and abdominal aortic calcification: Results of a nationwide survey. Nutri Metabol Cardiovasc Dis NMCD. (2023) 33(7):1437–43. doi: 10.1016/j.numecd.2023.04.015 37156667

[30] Bagyura Z Kiss L Lux Á Csobay-Novák C Jermendy ÁL Polgár L. Neutrophil-to-lymphocyte ratio is an independent risk factor for coronary artery disease in central obesity. Int J Mol Sci. (2023) 24. doi: 10.3390/ijms24087397 PMC10138538 37108560

[31] Sage AP Tintut Y Demer LL. Regulatory mechanisms in vascular calcification. Nat Rev Cardiol. (2010) 7:528–36. doi: 10.1038/nrcardio.2010.115 PMC3014092 20664518

[32] New SEP Aikawa E. Molecular imaging insights into early inflammatory stages of arterial and aortic valve calcification. Circ Res. (2011) 108:1381–91. doi: 10.1161/CIRCRESAHA.110.234146 PMC3139950 21617135

[33] Chapman CML Beilby JP McQuillan BM Thompson PL Hung J. Monocyte count, but not C-reactive protein or interleukin-6, is an independent risk marker for subclinical carotid atherosclerosis. Stroke. (2004) 35:1619–24. doi: 10.1161/01.STR.0000130857.19423.ad 15155967

[34] Celi A Pellegrini G Lorenzet R De Blasi A Ready N Furie BC. P-selectin induces the expression of tissue factor on monocytes. Proc Natl Acad Sci USA. (1994) 91:8767–71. doi: 10.1073/pnas.91.19.8767 PMC44687 7522321

[35] Martinod K Wagner DD. Thrombosis: tangled up in NETs. Blood. (2014) 123:2768–76. doi: 10.1182/blood-2013-10-463646 PMC4007606 24366358

[36] Li T Yu H Zhang D Feng T Miao M Li J. Matrix vesicles as a therapeutic target for vascular calcification. Front Cell Dev Biol. (2022) 10:825622. doi: 10.3389/fcell.2022.825622 35127686 PMC8814528

[37] Zazzeroni L Faggioli G Pasquinelli G. Mechanisms of arterial calcification: the role of matrix vesicles. Eur J Vasc Endovascul Surg. (2018) 55:425–32. doi: 10.1016/j.ejvs.2017.12.009 29371036

